# Bilateral adrenal enhancement revised—adrenal-to-spleen ratio as an appropriate mortality predictor

**DOI:** 10.1007/s00261-020-02886-9

**Published:** 2020-12-11

**Authors:** Robert Winzer, Ron Martin, Daniel Kaiser, Jan Christian Baldus, Sebastian Hoberück, Ralf-Thorsten Hoffmann, Dieter Fedders

**Affiliations:** 1 Department of Radiology, Dresden University Hospital, Fetscherstr. 74, 01307 Dresden, Germany; 2 Department of Neuroradiology, Dresden University Hospital, Fetscherstr. 74, 01307 Dresden, Germany; 3 Department of Nuclear Medicine, Dresden University Hospital, Fetscherstr. 74, 01307 Dresden, Germany

**Keywords:** Hospital mortality, Prognosis, Mortality prediction, Intensive care unit, Adrenal enhancement

## Abstract

**Purpose:**

To investigate whether adrenal gland radiodensities alone or set in relation to either the inferior vena cava (IVC) or the spleen can predict hospital mortality in intensive care unit patients.

**Methods:**

One hundred thirty-three intensive care patients (90 males, age: 66.3 ± 14.5 years) with an acute clinical deterioration were included in this retrospective analysis. CT attenuation (Hounsfield units) of adrenal glands, IVC, and spleen was evaluated by 2 radiologists separately. Adrenal-to-IVC and adrenal-to-spleen ratios were calculated. Receiver operating characteristic (ROC) analysis, combined with the Matthews correlation coefficient (MCC) as a classifier, was used to assess which parameter is the most suitable for short-term, intermediate-term, and overall mortality prediction. Interrater agreement was assessed using intraclass correlation coefficient (ICC).

**Results:**

The highest discriminative power to distinguish between deceased and survivors was found for the adrenal gland-to-spleen ratio for the 72-h mortality. A threshold of > 1.4 predicted 72-h mortality with a sensitivity of 79.31% and a specificity of 98.08% (area und the curve (AUC) = 0.94; *p* < 0.0001; MCCs = 0.81). The positive likelihood ratio was 41; the positive predictive value was 92.20%. Adrenal gland-to-spleen ratio was also best suited to predict the 24-h and overall mortality. ICCs of HU measurements in adrenal gland, IVC, and spleen indicated a high interrater agreement (ICC 0.95–0.99).

**Conclusions:**

To conclude, the adrenal-to-spleen ratio in CT in portal venous phase may serve as an imaged-based predictor for short, intermediate, and overall mortality and as reproducible prognostic marker for patient outcome.

## Introduction

The term CT hypoperfusion complex [1–5] includes various imaging features seen in the context of profound hypoperfusion and circulatory shock. In many patients with shock, the adrenal glands show intense enhancement as an imaging correlate of an increased release of catecholamines [6]. Catecholamines are crucial endogenous agents for increasing blood flow to the vital organs, which are typically triggered by a significant drop in blood pressure. By releasing these hormones, adrenal glands play a central role in the circulatory regulation in states of shock.

Several study groups defined adrenal enhancement as bilateral adrenal attenuation values higher than those of the inferior vena cava (IVC) [2–5, 7–11]. Boos et al. [10] and Schek et al. [11] showed that adrenal enhancement is associated with a poor outcome. However, Schek’s and Boos’ inclusion of the IVC in their definition of hyperattenuating raises questions about IVC’s suitability as a reference region. Due to pooling, e.g., in right ventricular failure with reflux of contrast agent into the inferior vena cava, and flow phenomena or the presence of catheters, it may be challenging to attain reproducible results in close-by regions or in repeated readings. Furthermore, no explanation for the choice of the IVC as reference region is given in any of the studies.

Lubner et al. [1] described that parenchymal abdominal organs such as the spleen may also be used as reference. The spleen may be suitable because of its high perfusion with a reservoir function for red blood cells and platelets and its specific organ answer in the event of a shock [12]. The sympathetic system’s activation leads to a reduced arterial inflow and an increased venous outflow [1]. This results in a decrease in volume and reduced density values following contrast medium application [13]. A decreased splenic enhancement in the arterial phase may be a useful image-based predictor of survival or death in patients with hypovolemic shock [14].

Therefore, this study aimed to investigate the suitability of parameters for adrenal gland radiodensities alone or in relation to the IVC or the spleen to predict mortality in intensive care unit patients.

Using the receiver operating characteristic (ROC) analysis, we determined thresholds for the short-term (24 h), intermediate-term (72 h), and overall mortality.

## Materials and methods

### Patients and study design

The institutional review board (EK 414092019) approved this retrospective study. We searched the radiology information system (RIS) of our institute for patients requiring intensive care treatment with a contrast-enhanced CT of the aorta between February 2019 and June 2019. Inclusion criteria were a CT with an unenhanced phase, a bolus-triggered arterial phase at the level of the diaphragm or in the descending aorta and a portal venous phase with complete imaging of the abdominal organs.

**Fig. 1:**
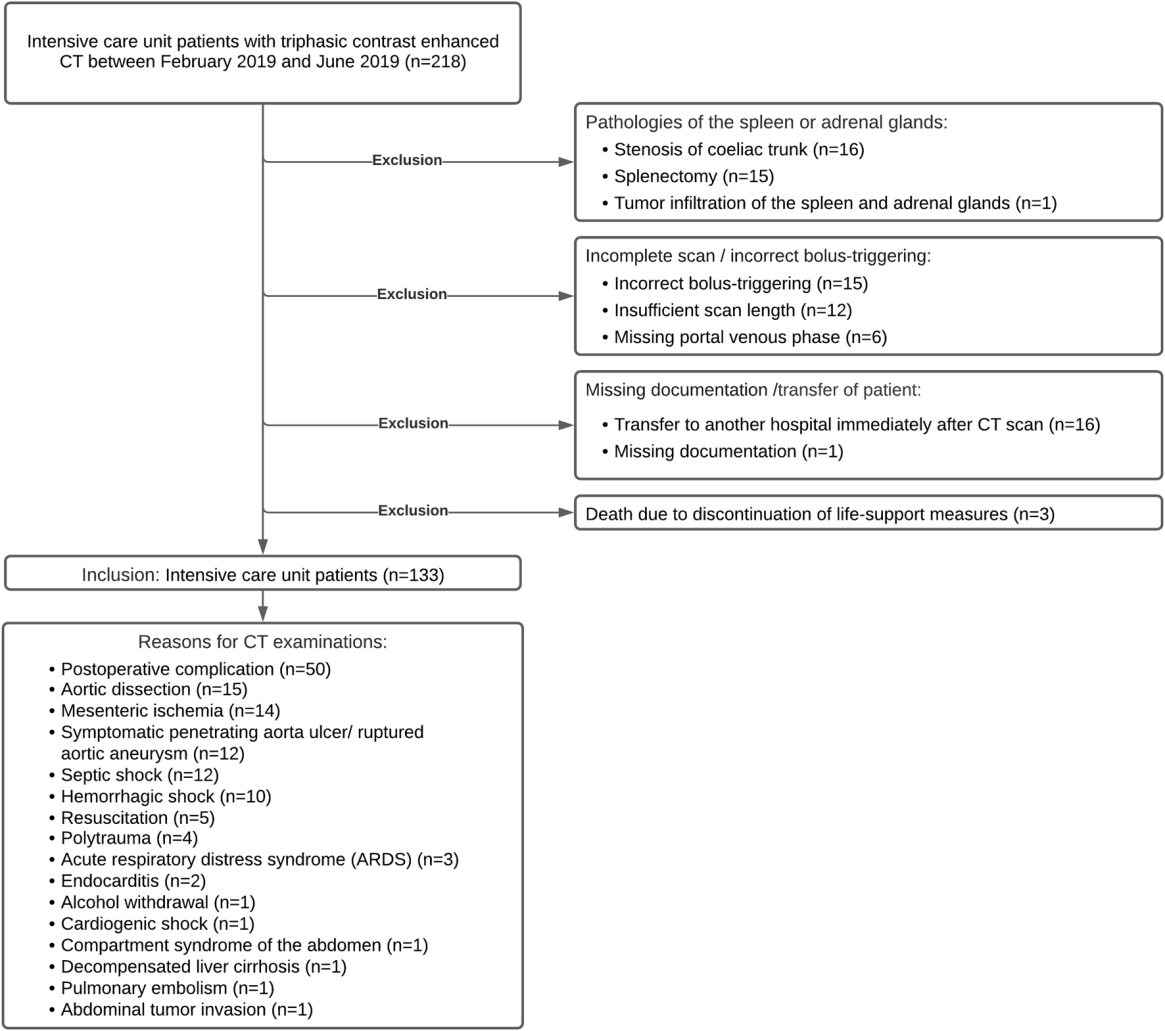
Study flow chart with numbers/reasons of included/excluded patients

This protocol is used at our institute for assessing acute and severe medical events such as bleeding or hemorrhagic shock. We excluded patients with adrenal hemorrhage, unilateral adrenal gland enhancement, higher grade stenosis of the coeliac trunk, splenic tumor infiltration, splenectomy, incomplete computer tomography, or missing clinical data (Fig. 1). Two hundred eighteen patients requiring intensive care received contrast-enhanced CT between February 2019 and June 2019. One hundred thirty-three of these patients (90 males, age: 66.3 ± 14.5 years) met the inclusion criteria. Reasons for the imaging and exclusion are listed in Fig. 1.

### CT image acquisition and post-processing

Patients underwent MDCT scanning on either a 64 slice scanner (Definition Sensation 64, Siemens, Forchheim, Germany) or a 2 × 192 slice scanner (Definition Somatom Force, Siemens, Forchheim, Germany). All examinations were performed at 110 kV with automated tube current modulation activated in all scans (CAREDose 4D, Siemens AG, Healthcare Section). Contrast attenuation was achieved using a bodyweight-adapted protocol for the injection of 1 ml/kg of non-ionic iodinated contrast medium (Ultravist 360 mgI/ml, Bayer Schering Pharma, Berlin, Germany or Solutrast 360 mgl/ml, Bracco Imaging Deutschland GmbH, Konstanz, Germany) at a flow rate of 3–4 ml/s, followed by a 50 ml saline flush. The arterial phase was started using bolus trigger technique with a threshold of 100 Hounsfield units in the descending aorta. Images of the native phase, the bolus-triggered arterial phase, and the portal venous phase—70 s after the start of injection—were acquired and archived with a slice thickness of 3 mm in our picture archiving and communication system (IMPAX Agfa HealthCare, Bonn, Germany).

### Image analysis

Two radiologists with 3 and 21 years of abdominal CT experience reviewed the CT examinations independently and blinded to patients’ clinical data. Image data evaluation was performed on a PACS workstation (IMPAX Agfa HealthCare, Bonn, Germany). Attenuation of the adrenal glands and IVC was analyzed on arterial and portal venous phase images on 3 mm slices. Window modulation was adapted by the radiologists individually. For quantitative analysis, radiodensities were determined preferably in the confluence of both adrenal limbs and in the IVC at the right adrenal gland level. Region-of-interest (ROI) measurements were used in axial CT images, as reported by previous studies [10]. All measurements were performed in greatly enlarged images to avoid adjacent fat. The preferred size of the ROI area was 30–40 mm^2^. The adrenal-to-IVC ratio was defined by the mean radiodensities of adrenal glands divided by the IVC’s radiodensity. The splenic attenuation value was obtained by placing three circular ROIs (2.0 cm^2^ in size) at three different axial levels through the superior third, middle third, and inferior third of the spleen in portal venous phase [15]. Hypodense triangular wedge-shaped areas at the periphery of the spleen were not included in the measurement. The averaged CT attenuation value was used for data analysis. The adrenal-to-spleen ratio was defined by the mean HU of adrenal glands divided by the averaged HU of the spleen.

### Outcome analysis

Death within 24 h and 72 h after imaging as well as overall mortality following the CT scan were set as the primary endpoint. After the measurements, clinical history from the electronic hospital information system (HIS) was matched. Diagnoses were obtained by reviewing the CT scans and clinical data.

### Statistical analysis

Statistical analysis was performed using MedCalc 16.8.0 (MedCalc Software bvba, Ostend, Belgium). Characteristics of the study population were given by means and standard deviations for continuous variables. For determining 24-h, 72-h, and overall mortality, we calculated thresholds based on ROC analysis for the absolute adrenal radiodensities, the adrenal-to-IVC, and the adrenal-to-spleen ratio and evaluated its suitability for predicting patients’ mortality. The area under the curve (AUC) was used as a measure of diagnostic accuracy [16], considering AUC < 0.5 as not useful, 0.5–0.6 as bad, 0.61–0.7 as sufficient, 0.71–0.8 as good, 0.81–0.9 as very good, and 0.91–1.0 as excellent regarding its discriminative power. Patients were classified by the calculated thresholds for the examined parameters mentioned above. Subsequently, the Matthews correlation coefficient (MCC) was calculated. MCC only produces a high score if the prediction obtained distinct results in all of the four confusion matrix categories (true positives, false positives, true negatives, and false negatives) [17–19]. A positive and negative likelihood ratio was presented for the highest MCC. Intraclass correlation coefficients were used to determine the interobserver agreement of adrenal gland, spleen, and IVC measurements [20]. Statistical significance was defined as *p* < 0.05.

## Results

### Patients’ outcome

**Fig. 2:**
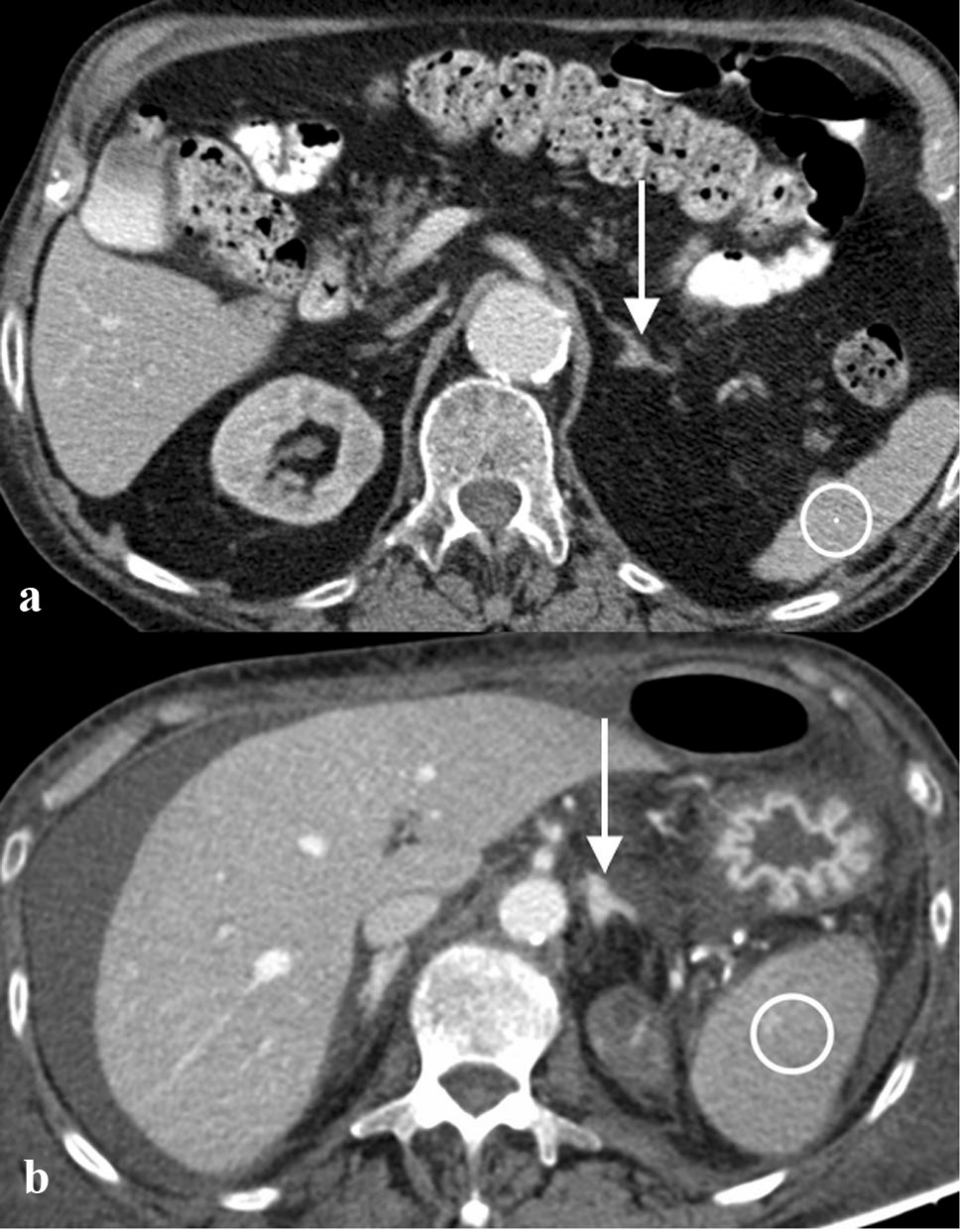
**a** Abdominal CT scan of an 83-year-old woman with aortic dissection of the ascending aorta. White-colored arrow point to the left hyperdense adrenal gland (average HU adrenal gland: 75); white-colored circle within the spleen (averaged HU spleen: 95; adrenal gland-to-spleen ratio  ≈ 0.79). The patient survived. **b** Abdominal CT scan of a 78-year-old male patient with septic shock. White-colored arrow point to the left hyperdense adrenal gland (average HU adrenal gland: 160); white-colored circle within the spleen (averaged HU spleen: 93; adrenal gland-to-spleen ratio: ≈ 1.72). The patient died within 24 h after the imaging

Twenty patients (15.0%) died within the first 24 h after the CT scan (Fig. 2). Within the first 72 h, 29 patients (21.8%) died. Another 9 patients died within 30 days after CT. The overall mortality rate was 28.6% (38 patients), and 95 patients survived.

### ROC analysis for the absolute adrenal gland radiodensities

Regarding mortality prediction, the evaluation of the absolute adrenal gland radiodensities in the portal venous phase demonstrated a high discriminative power (AUC 0.85–0.87). The best results for absolute adrenal gland radiodensities were found for the 72-hour mortality prediction in the portal venous phase. A cut-off value above 95 HU yielded a sensitivity of 82.76% (95% CI 64.2–94.2) and a specificity of 91.35% (95% CI 84.2–96.0) for the event to die within the first 72 h after CT (AUC = 0.87, *p* = 0.0001, MCC = 0.71). The positive predictive value was 72.7% (95% CI 58.3–83.6); the negative predictive value was 95.0% (95% CI 89.5–97.7). The positive likelihood ratio was 9.56 (95% CI 5.0–18.2). The negative likelihood ratio was 0.19 (95% CI 0.08–0.4). The post-test probability was approximately 72.7% under the assumption of a pre-test probability of 21.8% (72-h mortality rate).

### ROC analysis for the adrenal-to-IVC ratio

The adrenal-to-IVC ratio showed a good discriminative power for predicting patients’ mortality in the portal venous phase (AUC 0.71–0.80). The best results were found for 72-h mortality prediction. A cut-off ratio > 0.83 yielded a sensitivity of 68.97% (95% CI 49.2–84.7) and a specificity of 85.58% (95% CI 77.3–91.7) for the event to die within 72 h after imaging (AUC = 0.79, *p* < 0.0001, MCC = 0.51). The positive predictive value was 57.1% (95% CI 44.0–69.3); the negative predictive value was 90.8% (95% CI 85.1–94.5). The positive likelihood ratio was 4.78 (95% CI 2.8–8.1); the negative likelihood ratio was 0.36 (95% CI 0.2–0.6). The post-test probability was approximately 57.1% under the assumption of a pre-test probability of 21.8% (72-hour mortality rate). For an assumed adrenal-to-IVC ratio ≥ 1.0, low MCC scores (0.13 - 0.35) were found.

### ROC analysis for the averaged spleen radiodensities

Evaluation of the HU spleen in the arterial and portal venous phase showed good to very good discriminative power (AUC 0.74–0.90) with moderate to high sensitivities and specificities. The best results were found for the 72-h mortality prediction in the arterial phase. Here, a cut-off value less than or equal to 54.67 HU yielded a sensitivity of 82.76% (95% CI 64.2–94.2) and a specificity of 87.50% (95% CI 79.6–93.2) for the event to die within the first 72 h after imaging (AUC = 0.90; *p* < 0.0001; MCC = 0.64). Likelihood ratios were insufficient with a positive likelihood ratio of 6.62 (95% CI 3.9–11.3) and a negative likelihood ratio of 0.20 (95% CI 0.09–0.4). The positive predictive value was 64.9% (95% CI 52.0–75.9); the negative predictive value was 94.8% (95% CI 89.1–97.6). The post-test probability was approximately 65% under the assumption of a pre-test probability of 21.8% (72-h mortality rate).

### ROC analysis for the adrenal-to-spleen ratio

In terms of mortality prediction, the portal venous phase’s adrenal-to-spleen ratio has an excellent discriminative power to distinguish between deceased and survivors (AUC 0.93–0.94). A cut-off ratio > 1.41 yielded a sensitivity of 79.31% (95% CI 60.3–92.0) and a specificity of 98.08% (95% CI 93.2–99.8) for the event to die within the first 72 h (AUC = 0.94, *p* < 0.0001, MCC = 0.82, Table 1). For 72-h mortality, the positive likelihood ratio was 41.24 (95% CI 10.3–164.8), and the negative likelihood ratio was 0.21 (95% CI 0.1–0.4). The positive predictive value was 92.0% (95% CI 74.2–97.9); the negative predictive value was 94.4% (95% CI 89.3–97.2). The post-test probability was approximately 92% under the assumption of a pre-test probability of 21.8% (72-hour mortality rate).

**Table 1:** ROC analysis results with cut-off values for the adrenal-to-spleen ratio for 24-h, 72-h, and overall mortality in arterial and portal venous phase

Mortality	Cut-off	AUC	SENS (%)	SPEC (%)	p	PPV (%)	NPV (%)	MCC
Adrenal-to-spleen ratio measurements in the arterial phase								
24-h	> 1.67	0.83	45.00	96.46	< 0.0001	69.2	90.8	0.49
72-h	> 1.07	0.88	75.86	89.42	< 0.0001	66.7	93.0	0.62
Overall	> 1.07	0.84	68.42	92.63	< 0.0001	78.8	88.0	0.63
Adrenal-to-spleen ratio measurements in the portal venous phase								
24-h	> 1.54	0.93	85.00	94.69	< 0.0001	73.3	97.3	0.75
72-h	> 1.41	0.94	79.31	98.08	< 0.0001	92.0	94.4	0.82
Overall	> 1.16	0.93	63.16	98.95	< 0.0001	96.0	87.0	0.72

*HU* Hounsfield units, *AUC* area under the curve, *SENS* sensitivity, *SPEC* specificity, *PPV* positive predictive value, *NPV* negative predictive value, *MCC* Matthews correlation coefficient

### Bilateral enhancement of adrenal glands: Parameter comparison and reproducibility of measurements

**Fig. 3:**
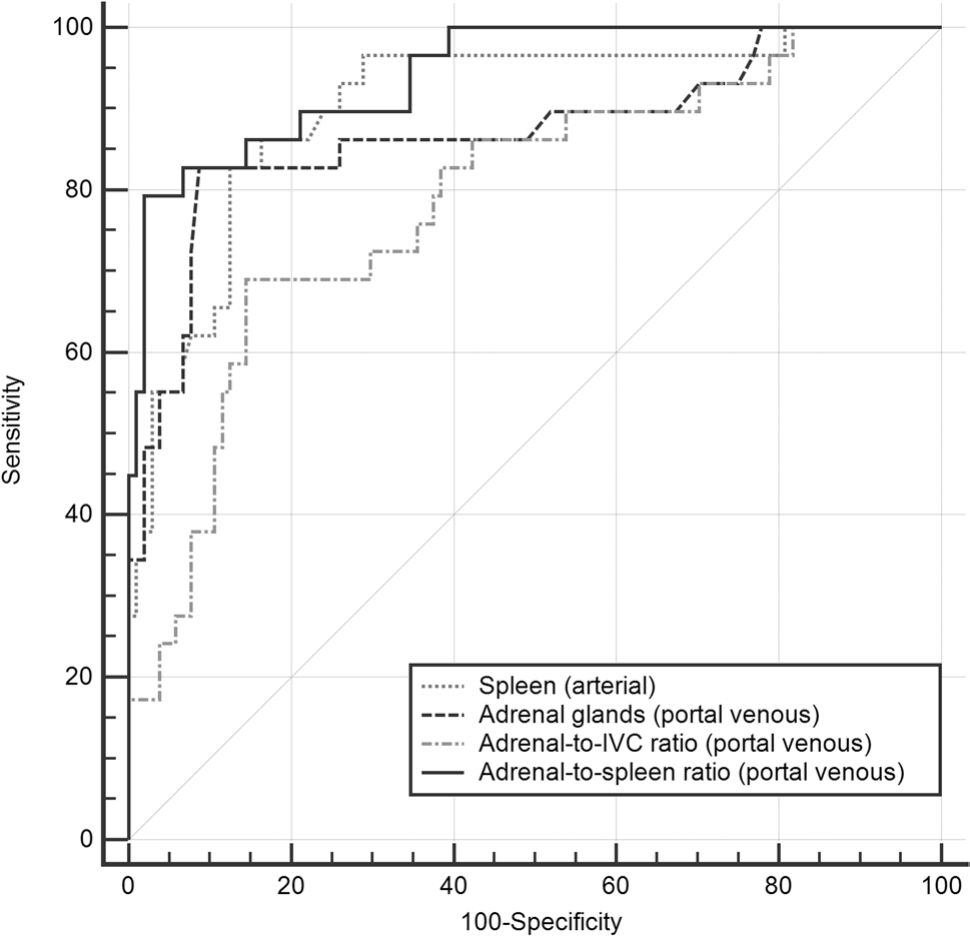
ROC curves comparison of best performing predictors for 72-h mortality. Highest area under the curve (= AUC) was found for adrenal-to-spleen ratio in portal venous phase (AUC = 0.94)

When comparing ROC curves for 24-h, 72-h, and overall mortality, the adrenal-to-spleen ratio (portal venous) was best suited for predicting mortality (Table 2 and Fig. 3). More important, the MCC—which only produces a high score if the prediction obtained distinct results in all of the four confusion matrix categories (true positives, false positives, true negatives, and false negatives)—for the adrenal-to-spleen ratio outperformed any other parameters in terms of mortality prediction (MCC_max_(*adrenal*-*to*-*spleen ratio) *= 0.82; MCC_max_(adrenal gland radiodensities*) *= 0.71; MCC_max_(adrenal-to-IVC ratio*) *= 0.51; MCC_max_(spleen radiodensities*) *= 0.64).

**Table 2:** Comparison of ROC curves for 24-h, 72-h, and overall mortality

	Adrenal glands (arterial)	Adrenal glands (venous)	Adrenal-to-IVC (arterial)	Adrenal-to-IVC (venous)	Adrenal-to-spleen-ratio (arterial)	Spleen (arterial)	Spleen (venous)
24-h mortality	AUC = 0.61	AUC = 0.87	AUC = 0.63	AUC = 0.79	AUC = 0.83	AUC = 0.89	AUC = 0.74
Adrenal-to-spleen ratio (venous) AUC = 0.93	0.32 < 0.0001	0.06 0.5933	0.30 < 0.0001	0.14 0.0073	0.1 0.0298	0.04 0.0431	0.19 0.0030
72-h mortality	AUC = 0.66	AUC = 0.87	AUC = 0.72	AUC = 0.79	AUC = 0.88	AUC = 0.90	AUC = 0.75
Adrenal-to-spleen ratio (venous) AUC = 0.94	0.28 < 0.0001	0.08 0.0515	0.18 0.0002	0.15 0.0002	0.06 0.1027	0.04 0.0931	0.19 0.0010
Overall mortality	AUC = 0.67	AUC = 0.85	AUC = 0.71	AUC = 0.81	AUC = 0.88	AUC = 0.87	AUC = 0.75
Adrenal-to-spleen ratio (venous) AUC = 0.93	0.26 < 0.0001	0.08 0.0103	0.22 < 0.0001	0.12 0.007	0.05 0.1085	0.06 0.0332	0.18 0.0001

When comparing all parameters, adrenal-to-spleen ratio (portal venous) was best suited for predicting the patients 24-h, 72-h, and overall mortality. Thus, we selected this parameter for AUC comparison with the other predictors. Data for differences between AUC and significance levels for comparison (*p*)

For measurements of adrenal glands, IVC, and spleen in the arterial and portal venous phases, interrater reliabilities were “excellent” (range 0.95–0.99).

## Discussion

This study’s objectives were to investigate whether bilateral adrenal enhancement either alone or in relation to the IVC or the spleen can predict hospital mortality in critically ill patients and which of the various parameters are best suited for this task. Our results suggest that the adrenal-to-spleen ratio in the portal venous phase is best suited to predict both the short-term to intermediate as well as the overall mortality of critically ill patients. The highest discriminative power was found for the portal venous adrenal-to-spleen ratio with an AUC of 0.94 for the 72-h mortality. Here, a cut-off ratio > 1.4 yielded a sensitivity around 80% and a specificity above 98% for the event of death within 72 h after CT scan. Absolute adrenal radiodensities in the portal venous phase can also be used to predict patients’ mortality with slightly lower power for prediction (MCC 0.64–0.71). As applied in previous studies [10, 11], the adrenal-to-IVC ratio was less suitable for mortality prediction, especially if a suggested adrenal-to-IVC ratio of 1 was applied.

To date, the prognostic value of the hyperdense adrenal glands for patients’ survival has only been studied to a limited extent. In the study by Boos’ et al. [10], “objective” hyperattenuating was defined as a difference between the adrenal glands and the IVC in the arterial phase of more than 15 HU. A parameter based on a relationship between the adrenal glands and the IVC in arterial phase has no apparent physiological background. Nevertheless, Boos’ et al. found a significantly higher rate of fatal outcomes in patients with hyperattenuating adrenal glands exceeding the radiodensity of the IVC in the described manner. For the venous phase, similar results were found in polytraumatized patients by Schek et al. [8]. Adrenal hyperattenuating was defined as an enhancement exceeding the radiodensities of the IVC. In our patient cohort, the adrenal-to-IVC ratio yielded only sufficient to good discriminatory qualities, depending on the contrast enhancement phase. The adrenal-to-IVC ROC analysis of radiodensities in the arterial phase showed only poor discriminating qualities expressed by a low MCC score and a low AUC. The MCC score and the AUC achieved better results in the portal venous phase. Besides, with a maximum sensitivity of 68%, the adrenal-to-IVC ratio is not suitable for screening. By increasing the cut-off ratio to 1, thus imitating the ratios used by Boos and Schek, specificity increased at the expense of sensitivity, rendering the adrenal-to-IVC ratio even less suitable for predicting mortality.

In contrast, the spleen seems to be a much better alternative for serving as a reference organ. In the event of a circulatory shock, the spleen fulfills its reservoir function by reducing the arterial inflow and increasing the venous outflow [1]. In this context, Enslow et al. demonstrated a decrease in volume and an associated reduction of the spleen’s density values [15]. Moreover, a decreased splenic enhancement in the arterial phase may be a mortality predictor in patients with hypovolemic shock [14]. This seems to be supported by our study results. The adrenal–spleen ratio takes these two phenomena with opposing directions into account, the decreasing density in the spleen and the increasing density of the adrenal glands. The adrenal–spleen ratio demonstrated an excellent discriminative power to distinguish between deceased and survivors (AUC 0.93–0.94) for all examined mortality periods. It may be suitable as an image-based screening parameter for critically ill patients by considerably increasing the post-test probability and due to its high positive predictive value. However, a smaller group of patients who also died during their further ICU stay, showed only slight changes in the adrenal glands and spleen radiodensities in the initial CT scan, resulting in a lower cut-off value for overall mortality.

Due to its retrospective study design, this study has some limitations. Furthermore, limitations may be attributed to measurement inaccuracies. Despite adequate zooming of the relatively small adrenals for measurement, adjacent adipose tissue and partial volume effects may have affected the results. When combining the spleen and adrenal gland measurements to form the portal venous adrenal-to-spleen ratio, the approach may be prone to error propagation. By measuring splenic attenuation at three different axial levels, excluding triangular wedge-shaped or inhomogeneous areas, and averaging the measurements, we achieved high interrater reliability (ICC 0.97) which may underline the reliability of the measurements. Still, subtle parenchymal diseases of the spleen still might be missed. The present study focused on comparing radiological parameters regarding their mortality prognosis ability. At this time, a correlation with clinical parameters was not evaluated. A prospective study should record an appropriate clinical- and serological-based assessment of multiple organ dysfunction simultaneously to correlate it with the adrenal gland enhancement as an image-based parameter. It is important to note that for other patient cohorts different thresholds may apply. Therefore, the validation of the determined cut-off values has to be investigated in further studies as well.

In about 15% of the initial patient group, technical reasons during image acquisition were responsible for their exclusion. Their exclusion was particularly necessary because of the limited assessment of the bolus-triggered arterial phase.

We chose the MCC because other discriminant measures for performance evaluation like accuracy, F1 score, and a classifier based on the Youden index can generate misleading results on imbalanced datasets. This is the case in our study; the number of surviving patients is much larger than the deceased. A classifier based on a high MCC score makes correct predictions both on the majority of the negative and the positive cases, independent of their total numbers in the dataset.

In critically ill patients, ROC analysis provided insights into the relation of different adrenal gland contrast enhancement parameters in different enhancement phases and the patient's risk to die within the next few days. The adrenal-to-spleen ratio ​​in the portal venous phase is best suited for short-term, intermediate-term, and overall mortality prediction.

The high positive likelihood ratios for the adrenal-to-spleen ratio in the portal venous phase indicate that for patients above a threshold of 1.4, the possibility of dying within 72 h was more than 40 times higher than for the patients below. Thus, the parameter seems to be well suited as an imaged-based screening instrument for critically ill patients. Since contrast-enhanced computed tomography is the method of choice for imaging patients with severe organ dysfunction, the analysis of the adrenal glands, and the spleen in the portal venous phase would be a by-product of imaging performed anyway. Patients with high adrenal density values in the portal venous phase (> 95 HU) or a high adrenal-to-spleen ratio (> 1.41) should get increased attention by the intensive care team.
